# Intérêt de l’enclouage centromédullaire dans les fractures du quart distal de la jambe: à propos de 30 cas

**DOI:** 10.11604/pamj.2017.28.176.10261

**Published:** 2017-10-25

**Authors:** Omar Margad, Jalal Boukhris, Hicham Sallahi, Ouahb Azriouil, Mohamed Daoudi, Khalid Koulali

**Affiliations:** 1 Service de Chirurgie Traumatologique et Orthopédique de l’Hôpital Militaire Avicenne, Marrakech, Maroc

**Keywords:** Fracture du quart inférieur, jambe, enclouage centromédullaire, plaque vissée, Fractures of the lower quarter, leg, centro-medullary nailing, screwed plate

## Abstract

Les fractures du quart distal de la jambe, sont des fractures dont le trait est situé au niveau du quart inférieur du tibia. Elles sont réputées graves et posent à la fois des problèmes de consolidation, de contention et de stabilité. Nous présentons l'expérience du service de traumatologie orthopédie de l'Hôpital militaire Avicenne de Marrakech, concernant 30 fractures fermées du quart inférieur de la jambe, sur une période de 10 ans (de Janvier 2001 à Décembre 2010) ayant reçu un enclouage centromédullaire. Le montage était verrouillé à 80% des cas et simple dans les autres cas. La moyenne d'âge de nos patients était de 36 ans. Il existait une nette prédominance masculine avec 27 hommes pour 3 femmes. La consolidation a été obtenue dans un délai normal de 17 semaines en moyenne et les résultats fonctionnels était satisfaisants. Un seul cas d'infection est survenu à 6 mois du geste chirurgical soit 3,3% et aucune autre complication n'a été mentionnée. Un cal vicieux a été retrouvé chez 30% de nos patients. Nos données épidémiologiques et nos résultats sont presque identiques à ceux de la littérature. Quant aux résultats angulaires, ils sont nettement inférieurs aux séries de plaques, à l'opposé, leurs résultats infectieux portent à la prudence et certains séries de clous rapportent d'excellents résultats angulaires à condition d'avoir un montage stable. A la lumière de ces résultats, nous sommes en droit de conclure au grand intérêt d'élargir les indications classiques de l'enclouage centromédullaire verrouillé à la prise en charge des fractures du quart distal de la jambe, à condition d'avoir un montage stable par un double verrouillage distal et une ostéosynthèse primaire des fractures distales de la fibula.

## Abstract (en)

The fractures of the distal quarter of the leg are characterized by fracture line located at the level of the lower quarter of the tibia, according to Gerard and Evrard definition [1]. They are serious and pose problems for consolidation, immobilization and stability. We here describe our experience in the Department of Orthopaedics and Traumatology at the Avicenne Military Hospital, Marrakech. We report 30 cases of closed fractures of the lower quarter of the leg treated with centro-medullary nailing over a period of 10 years (January 2001-December 2010). Locked nailing was performed in 80% of cases and simple nailing was performed in the other cases. The average age of patients was 36 years. There was a clear male predominance (27 men, 3 women). The average time for consolidation was 17 weeks and functional outcomes were satisfactory. A single case of infection occurred 6 months after surgery (3.3%) and no other complication was reported. Malunion was detected in 30% of patients. Our epidemiological data and results were almost identical to those in the literature. Angular results were significantly lower than those obtained with the series of plates. By contrast, data on infections called for caution and some nails produced excellent angular results when nail fixation was stable. In the light of these results, codified indications for locked centro-medullary nailing should be extended to the fractures of the lower quarter of the leg, provided that stable fixation using double screw distal locking and primary osteosynthesis of distal fibula fractures are performed.

## Introduction

Les fractures du quart distal de la jambe (FQDJ) sont rares. Par leur instabilité et leur situation anatomique, elles posent de véritables difficultés thérapeutiques. Le but de notre travail est d'étudier les caractères épidémiologiques et anatomopathologiques des FQDJ ainsi que déterminer la place de l'enclouage centromédullaire (l'ECM) pour le traitement de telles fractures.

## Méthodes

**Figure 1:**
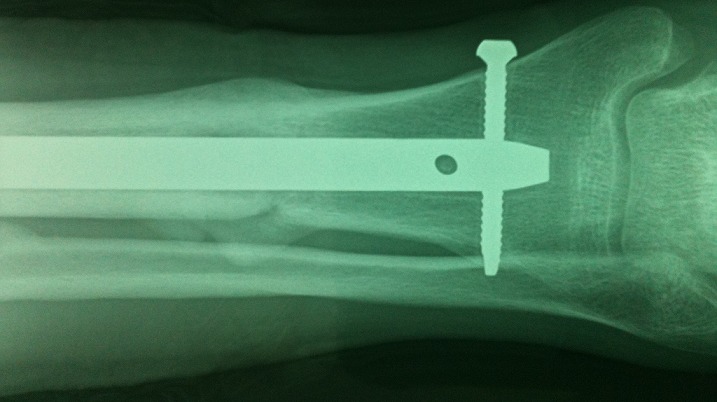
Fracture spiroïde du quart distal de la jambe classée A1-1 consolidée avec restitution de l’axe anatomique

Il s'agit d'une étude rétrospective s'étalant sur 10 ans entre 2001 et 2010 de 30 patients opérés pour FQDJ par ECM antérograde (Figure 1) à l'hôpital militaire Avicenne de Marrakech. Le quart distal correspondait au quart de la jambe calculée des épines tibiales au plafond de la mortaise tibio-talienne. Les critères d'exclusion comportaient les fractures ouvertes, avec extension articulaire, les fractures de l'enfant, sur os pathologique et les reprises chirurgicales suite à l'échec d'une autre méthode d'ostéosynthèse. Notre série de 30 patients comportait 27 hommes (90%) et 3 femmes (10%), d'âge moyen de 36 ans (20-51) au moment de l'accident. Le coté droit était atteint dans 17 cas (57%) et le coté gauche dans 13 cas (43%).les étiologies étaient comme suit: les accidents de la voie publique dans 13 cas soit 43%, les chutes dans 8 cas soit 27%, les accidents sportifs dans 4 cas (13%), les accidents de travail dans 3 cas (10%) et les agressions dans 2 cas (7%). La distance moyenne entre la fracture et la surface articulaire du pilon tibial était de 40 mm avec des extrêmes entre 32 et 60 mm. Les fractures ont été classées selon la classification de l'AO/OTA [**1**]: 20cas (67%) étaient classées A1, 6 cas (20%) A2 et 4 cas (13%) A3. Une fracture de la fibula était associée dans 25 cas (83%).tous nos patients ont eu une rachianesthésie, l'installation était en décubitus dorsal sur table ordinaire, jambe pendante verticale à l'aide d'une barre à genou. La réduction était manuelle sous contrôle scopique et l'ECM était réalisé avec alésage. Le montage était verrouillé dans 24 cas (80%), (dynamique dans 13 cas (43%), statique dans 11 cas (37%)), simple dans 6 cas (20%). Parmi les 25 fractures de la fibula seulement 2 fractures soit 8 % ont été opérées.

## Résultats

**Fonctionnels:** L'évaluation clinique devrait inclure le score d'Olerud et Molander, maismalheureusement, nos dossiers cliniques ne relevant pas de données à ce sujet. Nous avons évalué les amplitudes articulaires du genou et de la cheville. Le genou était normal dans tous les cas, la cheville dans 73% (22 patients). Un déficit de flexion dorsale a été noté chez 7 patients soit 24% (5 cas avec un déficit de 5° et 2 cas de 10°) et un seul cas (3%) avait un déficit de flexion plantaire de 5°. Pour les troubles rotationnels, on a eu 3 cas: un cas concernant la rotation interne < à 10°et deux cas, la rotation externe; un cas supérieur à 10° et l'autre inférieur à 10°. Enfin, l'appui partiel a été autorisé en moyenne à la 3^ème^ semaine, total en moyenne au 90°jour.

**Figure 2:**
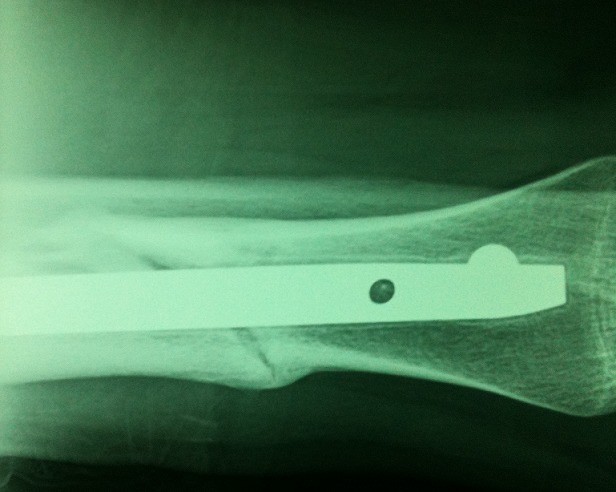
La même fracture vue de profil

**Radiologiques:** Le délai moyen de consolidation était de 17 semaines (15-33 semaines) (Figure 2). Nous avons enregistré un cas (3%) de retard de consolidation sur un montage simple qui a bien évolué après changement de clou avec un autre de diamètre plus élevé et avec un verrouillage statique. Aussi, on a eu un cas de pseudarthrose aseptique également sur un montage simple. L'évolution a été favorable après ablation d'un séquestre osseux, greffe cortico-spongieuse iliaque et verrouillage dynamique distal. Pour les résultats angulaires, on a eu 30% de cal vicieux, (un défaut d'axe supérieur à 5° était considéré comme cal vicieux).

**Complications:** On a observé un seul cas d'infection après 6 mois du geste chirurgical et après consolidation acquise. Il s'agissait d'un sepsis en regard de la vis de verrouillage distale bien évoluée après parage et antibiothérapie adaptée.

## Discussion

Les FQDJ sont reconnues rares et délicates, 0,7%pour Court -Brown et Caesar [1] ,10% pour Fan et al [2]. Cette rareté explique le nombre faible de fractures rapportées dans notre série.

**Analyse des données épidémiologiques et anatomopathologiques:** On comparant nos résultats avec les différentes séries de la littérature [3-5], nous remarquons que ce sont des fractures de l'adulte jeune caractérisées par une prédominance masculine et de l'atteinte du coté droit. L'étiologie la plus fréquente est les accidents de la voie publique. Les traits de fractures spiroïde sont en tête de liste, nous pouvons en déduire que le mécanisme le plus fréquent est un mécanisme de torsion. Ces fractures sont le plus souvent associées à une fracture de la fibula. La littérature rapporte un taux très réduit de fractures isolées de tibia compris entre 2% et 9%. Quant à notre série, ce taux est plus élevé et est de 17%. Nous n'avons pas inclu dans notre étude les fractures ouvertes ainsi que les fractures à extension articulaire et ce pour pouvoir étudier ces fractures dans les situations de base loin de tout risque de complication surajouté. Les revues rapportent un taux de fractures ouvertes compris entre 20% et 40%.Nous notons également la prédominance des fractures extra articulaires métaphysaires simples A1.

**Les résultats fonctionnels:** Les résultats de l'ECM et de l'ostéosynthèse par plaques vissées retrouvées dans la littérature [6-9] apparaissent globalement similaires avec des scores d'Olerud et Molander [10] voisins de 85%.

**Les résultats angulaires:** Notre taux de cal vicieux (30%) est bien supérieur à celui de la littérature [3,4]. Cette dernière rapporte comme facteurs de risque un défaut de réduction, l'élargissement métaphysaire, la comminution fracturaire, la technique chirurgicale avec un mauvais point d'entrée ou un mauvais positionnement du guide (qui doit être centré sur le profil et légèrement latéral sur la face), l'absence de la synthèse de la fibula et pour certains auteurs le jeune âge du patient. Nous retrouvons le taux le plus bas de cal vicieux qui est de 8% dans la série de la JBJS [4] .Ceci peut être expliqué par le montage verrouillé pouvant arriver jusqu'à 3 vis de verrouillage distaux et à l'ostéosynthèse primaire de la fibula qui est de 53% des cas, ce chiffre est le plus haut de la littérature.les résultats angulaires des séries de plaques [6-9] sont très prometteurs avec un taux bas de cal vicieux .Néanmoins, la série JBJS rapporte un taux bas de cal vicieux. Pour cette raison, l'ECM pourrait être un bon choix thérapeutique mais à condition de le réaliser dans les situations optimales.

**Les complications:** Nous rapportons un taux bas de retard de consolidation et de pseudarthrose par rapport aux autres séries de clous.les plaques vissées semblent à leur tour donner moins de retard de consolidation et de pseudarthrose. Néanmoins, les reprises chirurgicales qu'elles nécessitent sont plus complexes. En plus dans l'ECM, la dynamisation est un moyen simple et fiable donnant d'excellents résultats dans les retards de consolidation. Ailleurs l'ECM semble induire très peu d'infections, par contre, il expose à un risque élevé de rupture du matériel chose qui confirme la stabilité du montage par plaque vissée. Cependant, un verrouillage distal à 2 vis serait nécessaire pour promouvoir la stabilité du montage et rejoindre les résultats des séries de plaques.

## Conclusion

L'ECM semble être le meilleur moyen thérapeutique pour les FQDJ à condition que le fragment distal permette un verrouillage efficace, de préférence double. La synthèse de la fibula, surtout dans les fractures fibulaires distales semble nécessaire, car elle améliore significativement la stabilisation et la restitution anatomique de l'axe jambier. L'ostéosynthèse par plaque peut êtrepréférée à l'ECM dans les fractures trop basses et dans les refends articulaires complexes.

### Etat des connaissances actuelles sur le sujet

- Les fractures du quart distal de la jambe sont réputées graves et posent à la fois des problèmes de consolidation, de contention et de stabilité;
- Plusieurs méthodes thérapeutiques ont été décrites pour de telles fractures: enclouage centro- médullaire, plaques vissées.

### Contribution de notre étude à la connaissance

- Notre étude confirme les données épidémiologiques et anatomopathologiques décrites dans la littérature;
- L'enclouage centromédullaire semble être le meilleur moyen thérapeutique pour les fractures du quart distal de la jambe à condition qu'un certain nombre de critères soient respectés.

## Conflits d’intérêts

Les auteurs ne déclarent aucun conflit d'intérêts.
